# Research on the evolution of the Chinese urban biomedicine innovation network pattern: An analysis using multispatial scales

**DOI:** 10.3389/fpubh.2022.1036586

**Published:** 2022-11-14

**Authors:** Zhimin Ren, Jiaao Yu, Liping Qiu, Xuya Hong, Shaobin Wei, Haiyan Zhou, Xiao Hu, Xiaolei Zhang, Wei Zhang, Isaac Akpemah Bathuure, Qican Yang, Ning Su, Wei Lee, Xiaoping Wang, Hao Hu

**Affiliations:** ^1^School of MBA, Zhejiang Gongshang University, Hangzhou, China; ^2^London College of Communication, University of the Arts London, London, United Kingdom; ^3^Global Value Chain Research Center, Zhejiang Gongshang University, Hangzhou, China; ^4^Institute of Digital Economy and Green Development, Chifeng University, Chifeng, China; ^5^Cash Crop Workstation, Shangcheng Bureau of Agriculture and Rural Affairs, Xinyang, China; ^6^School of Economics and Management, Chifeng University, Chifeng, China; ^7^Academic Affairs Office, Xing'an Vocational and Technical College, Ulanhot, China; ^8^School of Urban and Regional Science, East China Normal University, Shanghai, China; ^9^College of Business Administration, Ningbo University of Finance and Economics, Ningbo, China; ^10^School of Economics, Shanghai University, Shanghai, China

**Keywords:** China, urban innovation network, biomedicine, network pattern, patent cooperation, multiscale

## Abstract

This paper addresses the spatial pattern of urban biomedicine innovation networks by separately using four scales, i.e., the national scale, interregional scale, urban agglomeration scale, and provincial scale, on the basis of Chinese biomedicine patent data from the incoPat global patent database (GPD) (2001–2020) and using the method of social network analysis (SNA). Through the research, it is found that (1) on the national scale, the Chinese biomedicine innovation network becomes denser from west to the east as its complexity continuously increases. Its spatial structure takes the form of a radial network pattern with Beijing and Shanghai as its centers. The COVID-19 pandemic has not had an obvious negative impact on this network at present. (2) On the interregional scale, the strength of interregional network ties is greater than that of intraregional network ties. The eastern, central and western biomedicine innovation networks appear to be heterogeneous networks with regional central cities as the cores. (3) At the urban agglomeration scale, the strength of intraurban-agglomeration network ties is greater than that of interurban-agglomeration network ties. The three major urban agglomerations have formed radial spatial patterns with central cities as the hubs. (4) At the provincial scale, the intraprovincial networks have poor connectivity and low internal ties strength, which manifest as core-periphery structures with the provincial capitals as centers. Our research conclusion helps to clarify the current accumulation of technology and offer guidance for the development of China's biomedicine industry.

## Introduction

The outbreak of the COVID-19 pandemic has accelerated the evolution and development of the biomedicine industry. As the “Diamond Industry” of the new century, biomedicine has already become a strategic industry in countries of the world, e.g., the biomedicine industry has been included in the high -end industrial field of national key development in China, and receives focused support by governments (1, 2). In light of the developing situation of the biomedicine industry at home and abroad, this sector favors spatially aggregated distribution, with a significant amount of clustering (3). At present, the Chinese biomedicine industry has taken on the notable characteristics of regional aggregation in the Yangtze River Delta (YRD), Beijing-Tianjin-Hebei (BTH) and Pearl River Delta (PRD) areas. With knowledge and information flowing faster across both individual regions and across the whole world, the biomedicine industry no longer seeks cooperative innovation within an agglomeration only and has rather begun to be characterized by interregional cooperation (4). With the support of policies, the Chinese biomedical industry has developed rapidly, but there are still some problems that restrict the further development of the industry, especially in regional innovation cooperation, which need to be solved. In practice, the means of seizing this opportunity to accelerate Chinese self-innovation in the biomedical industry and to optimize the spatial structure of this industry has become an urgent problem to be solved in postpandemic China.

In addition, since the 1980s, the research perspective regarding contemporary economic geography has shifted from that of “Relationship” to that of “Flow Space,” the traditional line mode has been transformed into a network mode, and networking innovation has become the mainstream of innovation research (5). In terms of methodology, most scholars have carried out research efforts on innovation networks by using data related to jointly applied patents and cooperative papers (6–8). With respect to research contents, scholars have conducted research and analysis of innovation networks on the basis of different disciplines and perspectives. In the early stages of this research, scholars studied the concepts and connotations of innovation networks mainly from perspectives such as organizational systems, information covenants, knowledge and skills, and regional space (9–15). To deepen this research, scholars have shifted their focus to the formation process, characteristics and structure of the innovation network using such SNA indices as centrality, structural hole, and network density, using knowledge flow, technological innovation, and industrial agglomeration, etc., as breakthrough points from industry perspectives such as those of bioscience and information technology (16–19).

Over recent years, scholars have generally attached greater importance to the factors influencing the formation of innovation networks, conducting research mainly on the internal structure of the network and its external environment. The internal structure of a network includes the general characteristics of the network, represented by indices such as network scale, and the network formation elements, represented by indices such as small world properties (19–24). Research on the external environment of a network mainly focuses on proximity and the regional environment where the subject is located (25–28).

A general review of the existing research finds that previous research efforts on innovation networks were mostly carried out on a single scale, e.g., the national scale, provincial scale, urban agglomeration scale, urban scale or rural scale (29–31). However, in existing research projects, investigations into urban innovation networks rarely take an urban perspective. Athey et al. (32) pointed out that research on innovation should take cities and the important characteristic of their orientation as basic units, since cities are geographic spaces where innovation subjects are the most active and centralized and are the places with the highest innovation efficiency. As a result, ascertaining the spatial situation of cooperation between Chinese biomedicine and Chinese biomedicine patent data can help to identify problems from a novel point of view and help to better understand China's technological accumulation in the biomedicine field.

The potential contributions of this research are as follows: (1) Taking cities as the basic unit, we assess the biomedical innovation capabilities of Chinese cities and deepen the research on urban innovation networks; (2) The research methods verticalize the original innovation network research perspective, namely, that of multispatial scales, and enrich the theoretical system of innovation geography; (3) Moreover, an in-depth analysis of the evolution of urban biomedicine innovation network patterns from the perspective of different spatial scales, e.g., the national scale, urban agglomeration scale, interregional scale, and provincial scale, will help drive the innovative growth of the Chinese biomedicine industry and offer guidance for the development of China's biomedicine industry.

The rest of this paper is organized as follows: Chapter 2 introduces the research methods and data sources; Chapter 3 analyzes the structure and evolution of China's urban biomedical innovation network from different spatial scales, e.g., the national scale, urban agglomeration scale, interregional scale, and provincial scale. Chapter 4 summarizes the research content and points out the potential contribution and limitations.

## Research data and methods

### Research data

The data quoted in this paper came from the incoPat GPD. The retrieval year interval spanned from 2001 to 2020, the retrieved objects were Chinese biomedicine patents (excluding H.K., Macao, and Taiwan), and the retrieval strategy was to require two or more applicants^1^ in the patent application, so a total of 37,350 biomedicine patents^2^ were selected.

### Research methods

In this paper, the SNA method was used to analyze the structural characteristics of Chinese urban biomedicine innovation networks and the evolution of their network patterns. The SNA method is a quantitative analysis method developed on the basis of mathematical methods, graph theory, etc., and it is one of the most widely used research methods in sociology and economics (33).

For our research, we defined overall network structures on four scales, i.e., the national scale, interregional scale, urban agglomeration scale, and provincial scale, by taking Chinese cities as the nodes of the network and the connections between the cities as the edges of the network. On this basis, we quantified the node, edge and overall characteristics of the urban biomedicine innovation network on multiple scales to study the evolution of the Chinese urban biomedicine innovation network pattern.

## Structural characteristics of the urban biomedicine innovation network on different spatiotemporal scales

### National scale

#### The Chinese national biomedicine innovation network becomes denser from west to east as its complexity continuously increases

**Figure 1:**
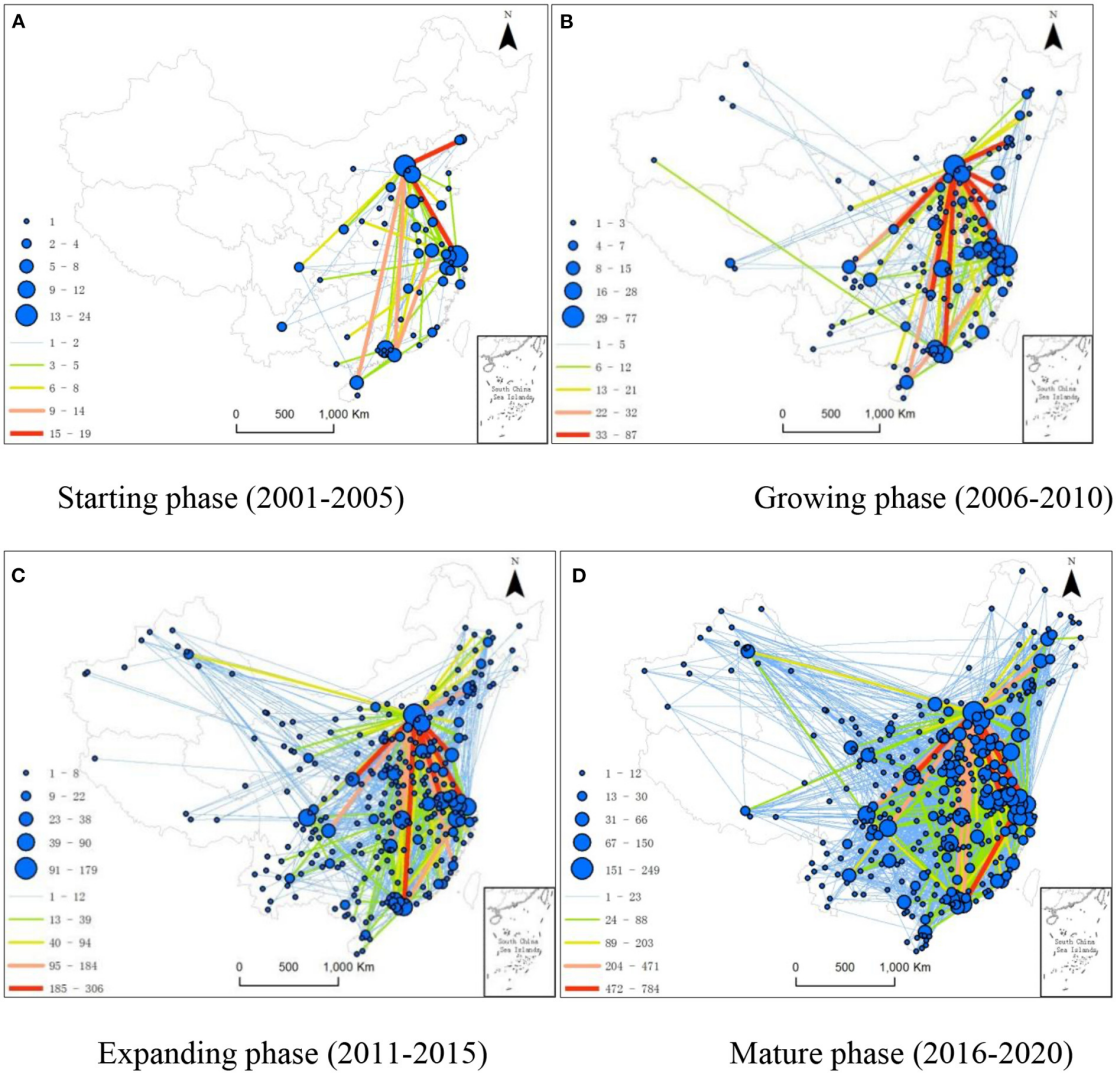
Spatiotemporal Evolution of the Chinese National Urban Biomedicine Innovation Network during the time span of 2001–2020. **(A)** Starting phase (2001–2005). **(B)** Growing phase (2006–2010). **(C)** Expanding phase (2011–2015). **(D)** Mature phase (2016–2020).

In this paper, the evolution process of the network was divided into four phases, namely, the starting phase (2001–2005), growing phase (2006–2010), expanding phase (2011–2015), and mature phase (2016–2020), according to the cooperative situation of urban biomedicine patents. In addition, the four phases of evolution of the Chinese urban biomedicine innovation network were visualized with ArcGIS software (Figure 1).

Starting phase: The 2001–2005 period is the starting phase of the Chinese national urban biomedicine network (Figure 1A). In this phase, the overall ties among the cities in the network were generally weak, and the network had a low density and a simple structure with a rare closed-loop innovation. In detail, less than one-fifth of the cities were brought into the biomedicine innovation network, with Beijing and Shanghai serving as the cores and Guangzhou and Tianjin as the main nodes. Chongqing, as a municipality controlled directly by the Central Government, did not exhibit an evident impetus toward radiating outwards during this phase. The ties between Beijing and Shanghai, Beijing and Fushun, Beijing and Shenzhen, Shenzhen and Nanjing, and Beijing and Haikou were all close. In terms of region, there were only six cities in the west that participated in the biomedical innovation network.

Growing phase: The 2006–1010 period represents the growing phase of the Chinese national urban biomedicine network (Figure 1B). In this phase, the network nodes increased considerably from those in the first phase, which indicates that there were more cities participating in the Chinese national urban biomedicine innovation network, which was experiencing an evidently expanding network size and a strengthening of the ties among cities. In terms of spatial structure, Beijing and Shanghai functioned as the radiation impetus center, with Nanjing, Guangzhou, Tianjin, Shenzhen, Hangzhou, and Wuhan serving as the main nodes. In detail, the density of the innovation network increased remarkably in the eastern coastal areas but remained at a low level in the west. Beijing and Shanghai continued to be the core cities and to play a radiation impetus role in the network during the growing phase. The ties between Beijing and Fushun and between Beijing and Shenzhen became increasingly close, which slightly differed from those in the starting phase. Meanwhile, some cities in the west were brought into the network, and the ties between the cities in the central region and those in the eastern region increased, with the interregional obstacles between the eastern and central regions gradually began to break down.

Expanding phase: The 2011–1015 period represents the expanding phase of the Chinese national urban biomedicine network (Figure 1C). In this phase, the number of cities participating in the biomedical innovation network increased by nearly 50% from that during the second phase, with the size of the network obviously expanding. The network in this phase exhibited a radial spatial structure with Beijing and Shanghai serving as the main radiation impetus cores and Nanjing, Guangzhou, Wuhan, Shenzhen, Hangzhou, Chengdu and Tianjin serving as the main nodes. The main ties in the network were between Beijing and Shanghai, Beijing and Tianjin, Beijing and Xi'an, Beijing and Jinan, Beijing and Qingdao, and Beijing and Shenzhen.

Mature phase: The 2016–1020 period represents the mature phase of the Chinese national urban biomedicine network (Figure 1D). During this phase, most Chinese cities were brought into the biomedicine innovation network, with both the size and density of the network increasing obviously. Beijing and Shanghai remained the core cities in this phase, and the network density in the west increased robustly. In particular, the radiation impetus abilities of Chengdu, Xi'an and Chongqing were enhanced, and Chengdu, Xi'an and Chongqing became the radiation impetus centers in the west, with close ties with the central and eastern regions. The tie strengths between Tianjin and Beijing, Beijing and Nanjing, Beijing and Xi'an, Beijing and Shanghai, Suzhou and Shenzhen, and Lianyungang and Shanghai were obviously strengthened compared with those in the expanding phase. In addition, many closed innovation loops, including Nanjing-Kunming-Baoshan, Beijing-Yulin-Xi'an, and Chongqing-Beijing-Yantai, were developed within the Chinese national urban biomedicine network. Such a closed network spatial structure enabled the flow of knowledge and information within the network to exhibit a self-reinforcing effect, which is helpful for biomedical innovation.

Generally, both the density and size of the Chinese national urban biomedicine innovation network have increased robustly, but the radiation impetus centers and innovation ties are all located in the eastern coastal areas, with the innovation network density becoming obviously lower as one moves west. In detail, Beijing and Shanghai have salient core positions in the innovation network with very strong radiation impetus abilities and the most extensive influence and scope. In all four phases, the innovation network displayed a radial spatial pattern with Beijing and Shanghai as the cores.

#### Overall ties in biomedicine innovation network strengthened over time, and polarization weakened

The statistical characteristics of the network (see Table 1) were assessed by calculating the nodes, edges, average degree and average weighted degree of the Chinese urban biomedicine innovation network throughout the 2001–2020 period using Gephi. In terms of network size, the numbers of nodes and edges increased from 56 and 88 in the 2001–2005 period to 337 and 2,889 in the 2016–2020 period, respectively, which indicates that in the mature phase, a total of 337 cities were brought into the national urban biomedicine innovation network; in other words, most Chinese cities had been incorporated into the network. In terms of network connectivity, the average degree rose from 3.14 in the 2001–2005 period to 17.15 in the 2016–2020 period, which means that on the national scale, in the mature period, every city had connected with ~17 other cities with respect to biomedicine patent cooperation, which accounted for ~5% of the total number of cities, indicating poor network connectivity. In terms of network ties strength, the average number of biomedicine patents related to intercity cooperation increased from 11 in the 2001–2005 period to 208 in the 2016–2020 period.

**Table 1:** Evolution of the statistical characteristics of the Chinese national urban biomedicine innovation network.

	**Period**	**Node**	**Edge**	**Average degree**	**Average weighted degree**
Entire network	2001–2005 period	56	88	3.14	11.11
	2006–2010 period	142	315	4.42	30.72
	2011–2015 period	280	1,080	7.71	83.64
	2016–2020 period	337	2,889	17.15	208.62
Ties strength >1	2001–2005 period	33	37	2.79	10.75
	2006–2010 period	89	280	3.94	30.24
	2011–2015 period	217	900	6.43	82.35
	2016–2020 period	303	2,165	12.85	204.33
Ties strength greater than the average	2001–2005 period	28	31	1.11	7.18
	2006–2010 period	55	80	1.13	22.44
	2011–2015 period	124	211	1.51	63.04
	2016–2020 period	172	463	2.75	160.74

The average values of cooperation strength in the 2001–2005 period, 2006–2010 period, 2011–2015 period, and 2016–2020 period were 3.53, 6.94, 10.84, and 12.17, respectively.

On the basis of the overall network properties and using hierarchic statistics, the data with tie strengths of >1 and those with tie strengths that were greater than the average were selected to form new networks, and then the nodes, edges, average degrees and average weighted degrees of the networks were calculated separately. When the tie strength was >1, either the number of nodes and edges or the average degree and average weighted degree decreased to a certain extent. However, the extent of such declines differed over time: the extent of the decline in the numbers of nodes and edges dropped from 41.07 and 57.95% in the 2001–2005 period to 10.09 and 25.06% in the 2016–2020 period, respectively. The extent of the decline in the average degree rose from 11.36% in the 2001–2005 period to 25.06% in the 2016–2020 period. This means that during the mature phase, by taking cooperation strength into account, we can see that the number of cities participating in the national biomedicine innovation network decreased by 34, and the intercity cooperation quantity decreased by 5, which is a large drop compared with that across the entire network. 10.09% of cities had an urban biomedicine innovation cooperation quantity of <1. Taking the average tie strength into account, the numbers of nodes and edges and the average degree of the network during the 2001–2005 period were 50.00, 35.23, and 35.22% of those across the entire network, respectively, and the numbers of nodes and edges and the average degree of the network in the 2016–2020 period were 51.04, 16.03, and 16.03% of those across the entire network, respectively. This means that over the 2001–2005 period, the technological cooperation quantity was less than the average in half of the cities, and ~65% of the biomedicine patent cooperation projects were located in 28 core cities, including Beijing, Shanghai, and Guangzhou, which had a salient polarization effect on the innovation network. In the 2016–2020 period, however, nearly half of the cities had biomedicine innovation cooperation quantities that were lower than the average, and nearly 80% of the patent cooperation projects were located in 172 cities, with the polarization effect within the innovation network greatly weakened.

Generally, the overall ties in the Chinese national urban biomedicine innovation network have been gradually strengthened, the cooperation scope has been somewhat expanded, and the polarization effect of the network has gradually disappeared.

#### Further discussion: Impact of the COVID-19 pandemic on the biomedicine innovation network

Investigating the Chinese urban biomedicine innovation network before and after the outbreak of the COVID-19 pandemic shows that the pandemic has not had a negative impact on Chinese biomedicine innovation cooperation. In contrast, the numbers of nodes and edges and the centrality and weighted centrality of the network have increased (see Table 2). The number of cities participating in the Chinese urban biomedicine innovation network increased by 19 from 2019 to 2020, with the number of network ties increasing by 298. In terms of network connectivity, the average degree increased from 9.26 in 2019 to 10.61 in 2020, which means that on the national scale, the number of cities cooperating with every other city regarding biomedicine patents increased from 9 to 10, and the network connectivity increased. It can be found from network ties strength that the average quantity of the intercity biomedicine patent cooperation projects rose from 57 in 2019 to 65 in 2020.

**Table 2:** Evolution of statistical characteristics and network patterns of the Chinese national urban biomedicine innovation network.

	**2019**	**2020**
Number of nodes	293	312
Number of edges	1,357	1,655
Centrality	9.26	10.61
Weighted centrality	57.07	65.61
Node (centrality)	Beijing (155) Shanghai (88) Nanjing (72) Wuhan (70) Guangzhou (70) Shenzhen (65) Chengdu (61) Hangzhou (58) Tianjin (55) Qingdao (54)	Beijing (179) Shanghai (104) Nanjing (83) Guangzhou (83) Wuhan (78) Xi'an (75) Hangzhou (71) Chengdu (67) Tianjin (60) Shenzhen (60)
Edge (weight)	Beijing-Tianjin (172) Shenzhen-Suzhou (172) Beijing-Shanghai (171) Beijing-Nanjing (155) Beijing-Wuhan (139) Beijing-Xi'an (127) Lianyungang-Shanghai (120) Beijing-Chengdu (116) Beijing-Dongying (109) Shanghai-Suzhou (106)	Beijing-Tianjin (253) Beijing-Xi'an (191) Beijing-Chengdu (162) Beijing-Nanjing (152) Shenzhen-Suzhou (130) Lianyungang-Shanghai (126) Beijing-Wuhan (119) Beijing-Qingdao (115) Shanghai-Suzhou (115) Beijing-Shanghai (111)

Beijing and Shanghai have served as the main Chinese innovation nodes and radiation impetus centers both before and after the outbreak of the COVID-19 pandemic. Qingdao dropped from the top 10 cities, and Xi'an replaced Qingdao and ranked 6th. In addition, among the top 10 nodes, the centrality of Shenzhen dropped from 65 in 2019 to 60, which means that the number of cities cooperating with Shenzhen in patents decreased to 60, while the centralities of other nodes increased to varying degrees. In 2019, the ties between Beijing and Tianjin, Shenzhen and Suzhou, Beijing and Shanghai, Beijing and Nanjing, and Beijing and Wuhan were the main ties in the network. In 2020, the ties between Beijing and Qingdao strengthened, ranking among the top 10.

### Interregional scale

#### Interregional network ties are stronger than intraregional network ties

For this research, China was divided into three regions, i.e., the eastern, central and western regions, and interregional urban biomedicine innovation networks were generated between each pair of regions. The basic statistical characteristics of the networks were calculated with the software Gephi to show the spatial structure properties of the Chinese interregional urban biomedicine innovation networks (see Table 3).

**Table 3:** Statistical characteristics of interregional urban biomedicine innovation networks among the eastern, central, and western regions of China.

**Region**	**Period**	**Number of nodes**	**Number of edges**	**Average degree**	**Average weighted degree**
Eastern–Eastern	2001–2005	39	63	3.23	12.31
	2006–2010	68	166	4.88	43.15
	2011–2015	97	425	8.76	140.95
	2016–2020	108	892	16.52	355.57
Central–Central	2001–2005	2	1	1.00	1.00
	2006–2010	20	16	1.60	3.30
	2011–2015	70	89	2.54	14.60
	2016–2020	106	272	5.13	33.98
Western–Western	2001–2005	0	0	0.00	0.00
	2006–2010	14	10	1.43	4.43
	2011–2015	55	68	2.47	11.75
	2016–2020	104	235	4.52	24.00
Eastern–Western	2001–2005	12	11	1.83	5.50
	2006–2010	38	49	2.58	14.74
	2011–2015	104	176	3.39	26.31
	2016–2020	176	522	5.93	57.69
Eastern–Central	2001–2005	17	13	1.53	4.35
	2006–2010	59	70	2.37	12.34
	2011–2015	137	271	3.96	36.10
	2016–2020	196	733	7.48	69.97
Central–Western	2001–2005	0	0	0.00	0.00
	2006–2010	4	3	1.50	3.00
	2011–2015	48	51	2.13	8.25
	2016–2020	120	235	3.92	16.15

From the statistical characteristics of the interregional urban biomedicine innovation networks, it can be seen that the sizes of these networks have evidently grown over time. The numbers of nodes between the eastern and central regions, between the eastern and western regions, and between the central and western regions rose from 17, 12, and 0 in the 2001–2005 period to 196, 176, and 120 in the 2016–2020 period, respectively, and the numbers of edges rose from 13, 11, and 0 in the 2001–2005 period to 733, 522, and 235 in the 2016–2020 period, respectively. In terms of network connectivity, the average degree of the networks rose from 1.53, 1.83, and 0 in the 2001–2005 period to 7.48, 5.93, and 3.92 in the 2016–2020 period, respectively, and the average weighted degree increased from 4.35, 5.50, and 0 in the 2001–2005 period to 69.97, 57.69, and 16.15 in the 2016–2020 period, respectively. As seen from the sizes of the intraregional urban biomedicine innovation networks, the numbers of nodes within the eastern, central and western regions in the 2016–2020 period rose by 1.77 times, 52 times and 104 times, respectively, compared with those in the 2001–2005 period, and the numbers of edges increased by 13.16 times, 271 times and 235 times, respectively. In terms of network connectivity, the average degrees in the networks rose by 5.08, 4.13 and 4.52 times, respectively, with the average weighted degrees increasing by 27.89, 32.98, and 24 times, respectively. In general, the interregional urban biomedicine innovation networks were superior in both tie closeness and strength to the intraregional urban biomedicine innovation networks, except for the intraregional urban biomedicine innovation network in the eastern region during the 2001–2010 period.

#### Heterogeneous space with regional central cities as cores formed in the eastern, central, and western regions

As seen from the spatial structure of the urban biomedicine innovation networks on an interregional scale (see Table 4), both the centrality of network nodes and the strength of the intercity biomedicine cooperation in the 2016–2020 period increased greatly compared with those in the 2001–2005 period, with an expansion of the heterogeneous space with regional central cities as cores that formed in the eastern, central and western regions.

**Table 4:** Spatial pattern of the Chinese urban biomedicine innovation network on the interregional scale in the 2016–2020 period.

**Region**	**Nodes (Top 5 in terms of centrality)**	**Edges (Top 5 in terms of weight. If the weights are more than 100, Top 10 are listed)**
Eastern–Eastern (2001–2005)	Beijing (17) Shanghai (14) Guangzhou (10) Tianjin (10) Nanjing (8)	Beijing-Shanghai (19) Beijing-Fushun (16) Beijing-Shenzhen (14) Shenzhen-Nanjing (13) Beijing-Haikou (10)
Eastern–Eastern (2016–2020)	Beijing (94) Shanghai (77) Nanjing (63) Guangzhou (61) Shenzhen (57)	Beijing-Tianjin (784) Beijing-Nanjing (659) Beijing-Shanghai (594) Shenzhen-Suzhou (587) Lianyungang-Shanghai (508) Beijing-Jinan (419) Beijing-Dongying (390) Shanghai-Suzhou (386) Beijing-Shenzhen (383) Beijing-Hangzhou (374)
Eastern–Central (2001–2005)	Beijing (4) Hefei (4) Shanghai (3) Jinan (2) Bengbu (1)	Yuncheng-Shanghai (8) Hefei-Shenzhen (7) Beijing-Hefei (3) Bengbu-Shanghai (2) Beijing-Jingzhou (2)
Eastern–Central (2016–2020)	Beijing (96) Wuhan (54) Shanghai (46) Nanjing (39) Zhengzhou (38)	Beijing-Wuhan (414) Beijing-Hefei (382) Beijing-Zhengzhou (367) Beijing-Changsha (294) Beijing-Changchun (277) Beijing-Puyang (144) Beijing-Daqing (142) Beijing-Nanchang (125) Shenzhen-Wuhan (108) Beijing-Huhhot (105)
Eastern–Western (2001–2005)	Chengdu (4) Shanghai (4) Beijing (3) Kunming (2) Xi'an (2)	Chengdu-Beijing (6) Liuzhou-Shanghai (6) Chongqing-Shanghai (4) Guangzhou-Fangchenggang (3) Xi'an-Beijing (3)
Eastern–Western (2016–2020)	Beijing (59) Xi'an (43) Chengdu (42) Chongqing (32) Nanjing (31)	Beijing-Xi'an (630) Beijing-Chengdu (471) Beijing-Chongqing (402) Beijing-Urumqi (174) Beijing-Yulin (162) Beijing-Kunming (120) Chengdu-Shenzhen (110) Kunming-Shanghai (86) Chengdu-Nanjing (80) Chengdu-Shanghai (77)
Central–Western (2001–2005)	None	None
Central–Western (2016–2020)	Wuhan (32) Xi'an (26) Chongqing (22) Chengdu (21) Changsha (19)	Chengdu-Wuhan (39) Zhengzhou-Chongqing (39) Wuhan-Chongqing (36) Xinxiang-Chongqing (33) Guigang-Wuhan (25)
Western–Western (2001–2005)	None	None
Western–Western (2016–2020)	Chengdu (36) Xi'an (30) Kunming (26) Chongqing (26) Nanning (21)	Chengdu-Mianyang (55) Chengdu-Chongqing (54) Chengdu-Xi'an (43) Xi'an-Xianyang (35) Chengdu-Urumqi (25)
Central–Central (2001–2005)	Yingtan (1) Nanchang (1)	Yingtan-Nanchang (1)
Central–Central (2016–2020)	Wuhan (41) Hefei (32) Changsha (29) Zhengzhou (26) Taiyuan (19)	Ezhou-Wuhan (82) Hefei-Zhengzhou (62) Jingzhou-Wuhan (54) Harbin-Jixin (53) Xinxiang-Zhengzhou (51)

It can be seen from the spatial structure in the 2016–2020 period that Beijing was the radiation impetus center of the eastern-central urban biomedicine innovation network, with Wuhan, Shanghai, Nanjing and Zhengzhou serving as subcenters. The main innovation ties in the network occurred between Beijing and cities in the central region, such as Wuhan and Hefei. The spatial structure of the eastern-eastern network is similar to that of the eastern-central network, with Beijing, Shanghai, Nanjing, Guangzhou and Shenzhen serving as important nodes. Many closed subnetworks were formed in the eastern-eastern urban biomedicine innovation network, and these subnetworks were located mainly in Nanjing, Shanghai, Suzhou and other cities in the YRD area and in Foshan, Guangzhou, Shenzhen and other cities in the PRD area.

The eastern-western urban biomedicine innovation network is relatively spatially expansive: in the northern part, four east–west axes are formed from Beijing as an apex to Xi'an, Chengdu, Chongqing and Urumqi in the western region; in the central part, two east–west axes are formed from Shanghai as an apex to Chengdu and Kunming in the western region; in the southern part, one east–west axis is formed between Shenzhen and Chengdu. Biomedical innovation ties are formed among the main nodes, and the distribution of the nodes within the network is relatively unbalanced. The central-western urban biomedicine innovation network exhibits a radial spatial structure with Wuhan as a radiation point that connects with Chengdu, Zhengzhou, and Chongqing. The western-central urban biomedicine innovation network basically spreads toward Wuhan, Xi'an and Chongqing, exhibiting a relatively significant imbalance. The spatial structure of the central-central network is relatively similar to that of the western-western network, with a maximum tie strength of no more than 90. The western-western urban biomedicine innovation network takes Chengdu, Xi'an, Kunming and Chongqing as cores, and the central-central urban biomedicine innovation network takes Wuhan, Hefei, Changsha and Zhengzhou, which are the provincial capitals of Hubei Province, Anhui Province, Hunan Province and Henan Province, respectively, as radiation points.

### Urban agglomeration scale

#### Intraurban-agglomeration network ties are stronger than interurban-agglomeration network ties

With three major Chinese urban agglomerations (BTH, YRD and PRD) being used as spatial units, the urban biomedicine innovation networks of BTH, YRD, PRD, PRD-BTH, PRD-YRD, and YRD-BTH arose separately. The basic statistical characteristics of the networks were calculated with the software Gephi to show the spatial and structural properties of Chinese interurban-agglomeration urban biomedicine innovation networks (see Table 5).

**Table 5:** Statistical characteristics of urban biomedicine innovation networks among BTH, YRD, and PRD.

**Period**	**Urban agglomeration**	**Number of nodes**	**Number of edges**	**Centrality**	**Weighted centrality**
2001–2005	BTH	4	4	2.00	5.50
2006–2010	BTH	8	10	2.50	30.25
2011–2015	BTH	11	19	3.46	119.27
2016–2020	BTH	13	36	5.54	296.15
2001–2005	PRD	5	4	1.60	3.60
2006–2010	PRD	7	7	2.00	10.00
2011–2015	PRD	9	16	3.56	69.11
2016–2020	PRD	9	21	4.67	266.67
2001–2005	YRD	12	14	2.33	7.17
2006–2010	YRD	18	39	4.33	42.67
2011–2015	YRD	25	83	6.64	92.48
2016–2020	YRD	26	141	10.85	263.08
2001–2005	PRD-BTH	4	4	2.00	13.00
2006–2010	PRD-BTH	8	8	2.00	32.25
2011–2015	PRD-BTH	13	13	2.00	66.00
2016–2020	PRD-BTH	16	26	3.25	128.00
2001–2005	PRD-YRD	6	5	1.67	9.33
2006–2010	PRD-YRD	12	11	1.83	10.00
2011–2015	PRD-YRD	22	39	3.55	45.27
2016–2020	PRD-YRD	32	79	4.94	107.44
2001–2005	YRD-BTH	9	9	2.00	9.56
2006–2010	YRD-BTH	18	20	2.22	22.78
2011–2015	YRD-BTH	32	39	2.44	73.31
2016–2020	YRD-BTH	38	80	4.21	176.21

As can be seen from the statistical characteristics of the urban biomedicine innovation networks among the three major Chinese urban agglomerations, the network sizes have increased over time, and all the cities that comprise the urban agglomerations participated in biomedicine innovation cooperation during the mature phase. Both the network connectivity and the network ties strength of an intraurban-agglomeration network are stronger than those of an interurban-agglomeration network. In terms of network connectivity, the intraurban-agglomeration network of the YRD has the strongest connectivity among the three major urban agglomerations. The interurban-agglomeration network between the PRD and YRD urban agglomerations has the strongest connectivity of the interurban-agglomeration networks. In terms of network tie strength, the intraurban-agglomeration network of BTH is the strongest among the three major urban agglomerations. The interurban agglomeration network between the YRD and BTH urban agglomerations has the strongest ties in the interurban agglomeration networks.

#### Radial spatial structure with central cities as hubs formed in three major urban agglomerations

As seen from the spatial structure of the urban biomedicine innovation networks on the urban agglomeration scale, both the centrality of network nodes and the intercity urban biomedicine cooperation strength were greatly increased in the 2016–2020 period compared with those in the 2001–2005 period, while the radial network structure using the central cities of the urban agglomerations as cores was enhanced.

The biomedical innovation networks of BTH, the YRD and the PRD have developed over the 2001–2005 period from single-core urban agglomerations with Beijing, Shanghai and Guangzhou serving as those single cores, respectively, to double-core urban agglomerations using Beijing and Shijiazhuang, Nanjing and Shanghai, and Guangzhou and Shenzhen as double cores, respectively (see Table 6). In the biomedical innovation network within the PRD urban agglomeration, cooperation was mainly carried out by Guangzhou with other cities, including Shenzhen and Foshan. In the biomedical innovation network within the YRD urban agglomeration, cooperation was mainly carried out by Nanjing and Shanghai with other cities, including Suzhou and Hangzhou. In the network within the BTH urban agglomeration, cooperation was mainly carried out by Beijing with other cities, including Shijiazhuang and Tianjin.

**Table 6:** Spatial pattern of Chinese urban biomedicine innovation networks on the interregional scale in the 2016–2020 period.

**Region**	**Nodes (Top 5 in terms of centrality)**	**Edges (Top 5 in terms of weight. If the weights are more than 100, then the Top 10 are listed)**
2001–2005 The PRD	Guangzhou (4) Foshan (1) Dongguan (1) Jiangmen (1) Shenzhen (1)	Guangzhou-Shenzhen (4) Fosha-Guangzhou (2) Guangzhou-Jiangmen (2) Guangzhou-Dongguan (1)
2016–2020 The PRD	Guangzhou (8) Shenzhen (8) Dongguan (6) Foshan (5) Zhuhai (4)	Guangzhou-Shenzhen (295) Foshan-Guangzhou (203) Guangzhou-Zhaoqing (202) Dongguang-Guangzhou (113) Guangzhou-Zhuhai (112)
2001–2005 The YRD	Shanghai (7) Hangzhou (4) Nanjing (4) Shaoxing (3) Jinhua (2)	Shanghai-Suzhou (7) Shanghai-Shaoxing (6) Hangzhou-Jinhua (5) Nanjing-Jiaxing (4) Taizhou-Shanghai (4)
2016–2020 The YRD	Nanjing (23) Shanghai (21) Hefei (20) Hangzhou (19) Suzhou (18)	Shanghai-Suzhou (386) Nanjing-Shanghai (196) Hangzhou-Shanghai (148) Nanjing-Suzhou (142) Shanghai-Taizhou (131) Shanghai-Shaoxing (111) Nantong-Shanghai (107)
2001–2005 The BTH	Beijing (3) Shijiazhuang (2) Tianjin (2) Langfang (1)	Beijing-Shijiazhuang (4) Beijing-Tianjin (4) Beijing-Langfang (2) Tianjin-Shijiazhuang (1)
2016–2020 The BTH	Beijing (12) Shijiazhuang (12) Tianjin (9) Baoding (7) Tangshan (5)	Beijing-Tianjin (784) Beijing-Shijiazhuang (328) Beijing-Langfang (275) Baoding-Beijing (148) Baoding-Shijiazhuang (59)
2001–2005 The PRD-The YRD	Hefei (2) Guangzhou (2) Shenzhen (2) Nanjing (2) Shanghai (1)	Shenzhen-Nanjing (13) Hefei-Shenzhen (7) Shanghai-Guangzhou (5) Zhaoqing-Nanjing (2) Hefei-Guangzhou (1)
2016–2020 The PRD-The YRD	Guangzhou (19) Shenzhen (17) Zhuhai (10) Shanghai (8) Zhongshan (8)	Shenzhen-Suzhou (587) Guangzhou-Shanghai (154) Shanghai-Shenzhen (99) Nanjing-Shenzhen (68) Guangzhou-Nanjing (63)
2001–2005 The PRD-The BTH	Beijing (2) Guangzhou (2) Shenzhen (2) Tianjin (2)	Beijing-Shenzhen (14) Guangzhou-Tianjin (7) Beijing-Guangzhou (3) Tianjin-Shenzhen (2)
2016–2020 The PRD-The BTH	Beijing (9) Shenzhen (6) Guangzhou (6) Tianjin (5) Zhuhai (4)	Beijing-Shenzhen (383) Beijing-Guangzhou (278) Guangzhou-Tianjin (59) Beijing-Dongguan (56) Beijing-Zhuhai (42)
2001–2005 The YRD-The BTH	Beijing (5) Shanghai (3) Tianjin (3) Suzhou (2) Hangzhou (1)	Beijing-Shanghai (19) Beijing-Hangzhou (4) Beijing-Wuxi (4) Suzhou-Tianjin (4) Xingtai-Shanghai (4)
2016–2020 The YRD-The BTH	Beijing (26) Tianjin (16) Shanghai (11) Nanjing (9) Shijiazhuang (8)	Beijing-Nanjing (659) Beijing-Shanghai (594) Beijing-Hefei (382) Beijing-Hangzhou (374) Beijing-Suzhou (185) Beijing-Jinhua (132)

In the interurban-agglomeration biomedicine innovation cooperation, the PRD-YRD had a double core structure with Guangzhou and Shenzhen as the cores, and both the PRD-BTH and the YRD-BTH used a single-core structure with Beijing as the single core. In the interurban-agglomeration biomedicine innovation cooperation network between the PRD and the YRD urban agglomerations, the level of cooperation between Guangzhou and Shenzhen in the PRD and between Shanghai and Suzhou in the YRD was dominant. In the interurban-agglomeration biomedicine innovation cooperation network between the PRD and BTH, the level of cooperation between Beijing in the BTH and Guangzhou and Shenzhen in the PRD was dominant. In the interurban-agglomeration biomedicine innovation cooperation network between the YRD and the BTH, the cooperation between Beijing in the BTH and Nanjing and Shanghai in the YRD was dominated.

### Provincial scale

#### Intraprovincial biomedical innovation networks have poor connectivity and low internal tie strength

The intraprovincial urban biomedicine innovation networks in 27 provinces, including Shaanxi, Shandong, and Guangdong, over the 2016–2020 period were investigated from the perspective of provincial scale (see Table 7). The network size of the urban biomedical innovation network in each province is small. In terms of network connectivity, the average degrees differ greatly among the provinces: the average degrees of eight provinces, i.e., Jiangsu, Shandong, Guangdong, Zhejiang, Fujian, Sichuan, Anhui, and Henan, are 3.05 higher than the average value, indicating a relatively high level of connectivity among the node cities within the network. The average degrees of Heilongjiang, Hainan, Ningxia and Tibet are lower than 2.0, while those of the other provinces are within the range of 2.0–3.0. In general, the connectivity among cities in the networks is not strong, with insufficient local networking. In the entire country, the average intraprovincial ties strength made up less than one tenth of the average strength of the total ties, which means that compared with interprovincial ties, the intraprovincial ties were relatively weak.

**Table 7:** Statistical characteristics of the intraprovincial urban biomedicine innovation networks of All Chinese Provinces in the 2016–2020 period.

**Province**	**Number** **of nodes**	**Number** **of edges**	**Average** **degree**	**Average** **weighted degree**	**Proportion of** **intraprovincial ties (%)**	**Province**	**Number** **of nodes**	**Number** **of edges**	**Average** **degree**	**Average** **weighted degree**	**Proportion of** **intraprovincial ties (%)**
Anhui	16	30	3.75	23.13	0.119	Jiangxi	6	6	2.00	30.00	0.052
Fujian	9	17	3.78	54.22	0.092	Liaoning	10	11	2.20	8.00	0.062
Gansu	9	10	2.22	10.67	0.112	Inner Mongolia	10	11	2.20	6.40	0.093
Guangdong	21	61	5.81	174.95	0.114	Ningxia	3	2	1.33	5.33	0.043
Guangxi	13	16	2.46	13.69	0.119	Qinghai	4	4	2.00	4.50	0.100
Guizhou	7	8	2.29	11.43	0.099	Shandong	17	54	6.35	96.35	0.117
Hainan	6	5	1.67	4.67	0.086	Shanxi	10	14	2.80	14.60	0.113
Hebei	11	16	2.91	26.73	0.077	Shanxi	9	10	2.22	17.78	0.060
Henan	17	29	3.41	33.06	0.104	Sichuan	17	32	3.77	25.29	0.116
Heilongjiang	9	8	1.78	16.44	0.079	Tibet	2	1	1.00	1.00	0.025
Hubei	15	19	2.53	39.73	0.082	Xinjiang	13	15	2.31	8.46	0.133
Hunan	12	16	2.67	21.17	0.088	Yunnan	16	24	3.00	15.25	0.156
Jilin	7	9	2.57	14.29	0.087	Zhejiang	11	29	5.27	103.09	0.088
Jiangsu	13	53	8.15	183.54	0.089						

#### The intraprovincial biomedicine innovation network forms a core-periphery structure with a provincial capital as the core

Table 8 shows that the provinces with multicore urban biomedical innovation networks include Shandong (with Jinan, Qingdao, and Yantai as the main nodes), Zhejiang (with Hangzhou, Ningbo and Shaoxing as the main nodes), Jiangsu (with Nanjing, Suzhou, Wuxi, and Yangzhou as the main nodes), and Fujian (with Fuzhou, Xiamen, and Quanzhou as the main nodes). Guangdong (with Guangzhou and Shenzhen as the main nodes) has a double-core network. Qinghai and Tibet only form biomedicine innovation ties between Haidong-Xining and Lhasa-Linzhi, respectively. Most of the other provinces use a core-periphery structure with the provincial capital as the core. The provinces with intraprovincial tie strengths of >90 include Guangdong, Jiangsu and Shandong, which means that the intraprovincial tie strengths in these provinces are relatively high. The provinces with intraprovincial tie strengths of <10 include Gansu, Hainan, Liaoning, Inner Mongolia, Ningxia, Qinghai, and Tibet, indicating that the intraprovincial tie strengths in these provinces are relatively low.

**Table 8:** Statistical characteristics of the intra-provincial urban biomedicine innovation networks of all Chinese provinces in the 2016–2020 period.

**Province**	**Nodes (Centrality)**	**Edges (Weight)**
Anhui	Hefei (15) Huaibei (6) Huainan (5) Fuyang (4) Chuzhou (4)	Hefei-Tongling (28) Hefei-Huaibei (25) Hefei-Huainan (16) Fuyang-Hefei (15) Chuzhou-Hefei (10)
Fujian	Fuzhou (8) Xiamen (6) Zhangzhou (5) Quanzhou (4) Longyan (3)	Fuzhou-Xiamen (40) Fuzhou-Sanming (34) Xiamen-Zhangzhou (31) Quanzhou-Xiamen (28) Fuzhou-Ningde (24)
Gansu	Lanzhou (8) Baiyin (3) Dingxi (2) Zhangye (2) Jinchang (1)	Baiyin-Lanzhou (9) Jiuquan-Lanzhou (9) Dingxi-Lanzhou (6) Lanzhou-Zhangye (6) Jinchang-Lanzhou (5)
Guangdong	Guangzhou (20) Shenzhen (18) Dongguan (10) Foshan (10) Zhanjiang (6)	Guangzhou-Shenzhen (295) Foshan-Guangzhou (203) Guangzhou-Zhaoqing (202) Dongguan-Guangzhou (113) Guangzhou-Zhuhai (112)
Guangxi	Nanjing (12) Laibin (3) Guilin (3) Baise (2) Hezhou (2)	Laibin-Nanning (11) Beihai-Nanning (10) Nanning-Baise (10) Nanning-Guilin (9) Nanning-Qinzhou (9)
Guizhou	Guiyang (6) Qiannanzhou (3) Anshun (2) Bijie (2) Tongren (1)	Guiyang-Qianxinanzhou (10) Bijie-Guiyang (8) Guiyang-Zunyi (7) Anshun-Guiyang (6) Bijie-Anshun (2)
Hainan	Haikou (5) Chengmai (1) Danzhou (1) Ding'an (1) Sanya (1)	Haikou-Sanya (6) Chengmai-Haikou (3) Danzhou-Haikou (2) Ding'an-Haikou (2) Lingshui-Haikou (1)
Hebei	Shijiazhuang (10) Baoding (5) Tangshan (3) Cangzhou (2) Hengshui (2)	Baoding-Shijiazhuang (59) Langfang-Shijiazhuang (37) Cangzhou-Shijiazhuang (9) Shijiazhuang-Qinhuangdao (9) Shijiazhuang-Tangshan (6)
Henan	Zhengzhou (15) Xinxiang (5) Xuchang (5) Pingdingshan (4) Anyang (3)	Xinxiang-Zhengzhou (51) Sanmenxia-Zhengzhou (41) Luoyang-Zhengzhou (39) Anyang-Zhengzhou (21) Nanyang-Zhengzhou (20)
Heilongjiang	Harbin (8) Daqing (1) Jixi (1) Jiamusi (1) Mudanjiang (1)	Harbin-Jixi (53) Suihua-Harbin (9) Daqing-Harbin (4) Harbin-Mudanjiang (3) Harbin-Qiqiha'er (2)
Hubei	Wuhan (14) Enshi (3) Yichang (3) Jingmen (2) Jingzhou (2)	Ezhou-Wuhan (82) Jingzhou-Wuhan (54) Huanggang-Wuhan (32) Wuhan-Xiaogan (22) Jingmen-Wuhan (19)
Hunan	Changsha (11) Xiangtan (4) Changde (3) Zhuzhou (3) Xiangxi (3)	Changde-Zhuzhou (38) Huaihua-Changsha (25) Xiangtan-Changsha (16) Changde-Changsha (9) Yueyang-Changsha (8)
Jilin	Changchun (6) Yanbian (4) Tonghua (3) Jilin (2) Siping (1)	Jilin-Tonghua (10) Jilin-Changchun (9) Yanbian-Changchun (8) Changchun-Tonghua (7) Siping-Changchun (6)
Jiangsu	Nanjing (12) Suzhou (12) Wuxi (11) Yangzhou (11) Zhenjiang (9)	Nanjing-Suzhou (142) Nanjing-Taizhou (95) Lianyungang-Nanjing (91) Nanjing-Wuxi (87) Changzhou-Nanjing (74)
Jiangxi	Nanchang (5) Ganzhou (2) Shangrao (2) Ji'an (1) Jiujiang (1)	Nanchang-Yichun (48) Nanchang-Jiujiang (19) Ganzhou-Nanchang (8) Ji'an-Nanchang (6) Ganzhou-Shangrao (5)
Liaoning	Shenyang (8) Dalian (4) Benxi (2) Jinzhou (2) Anshan (1)	Dalian-Shenyang (8) Dalian-Jinzhou (6) Shenyang-Tieling (5) Fushun-Shenyang (4) Liaoyang-Shenyang (4)
Inner Mongolia	Huhhot (9) Hinggan League (3) Chifeng (2) Tongliao (2) Bayannur (1)	Huhhot-Hinggan League (8) Huhhot-Xilingol League (5) Hinggan League-Hinggan League (4) Huhhot-Chifeng (3) Huhhot-Ulanqab (3)
Ningxia	Yinchuan (2) Wuzhong (1) Guyuan (1)	Wuzhong-Yinchuan (7) Yinchuan-Guyuan (1)
Qinghai	Haixi (1) Xining (1)	Haidong-Xining (6)
Shandong	Jinan (15) Qingdao (12) Yantai (10) Tai'an (8) Zibo (8)	Qingdao-Weifang (116) Jinan-Qingdao (71) Dezhou-Jinan (52) Jinan-Taian (51) Jinan-Jining (40)
Shanxi	Taiyuan (9) Jinzhong (5) Linfen (3) Lvliang (3) Jincheng (2)	Jinzhong-Taiyuan (34) Taiyuan-Xinzhou (7) Taiyuan-Changzhi (7) Taiyuan-Yuncheng (5) Linfen-Taiyuan (3)
Shaanxi	Xi'an (8) Weinan (3) Ankang (2) Xianyang (2) Baoji (1)	Xi'an-Xianyang (35) Xi'an-Yulin (11) Baoji-Xi'an (9) Hanzhong-Xi'an (9) Xi'an-Ankang (7)
Sichuan	Chengdu (16) Liangshan (7) Yibin (7) Luzhou (6) Panzhihua (6)	Chengdu-Mianyang (55) Chengdu-Panzhihua (21) Chengdu-Meishan (15) Chengdu-Zigong (14) Chengdu-Leshan (13)
Tibet	Lhasa (1) Linzhi (1)	Lhasa-Linzhi (1)
Xinjiang	Ili (4) Changji (3) Bayingolin (2) Shihezi (2) Aksu	Urumqi-Ili Kazak Autonomous Prefecture (13) Changji Hui Autonomous Prefecture-Urumqi (10) Turpan-Urumqi (6) Bayingolin Mongol Autonomous Prefecture-Urumqi (4) Shihezi-Ili Kazak Autonomous Prefecture (3)
Yunnan	Kunming (15) Chuxiong (5) Xishuangbanna (4) Honghe (4) Dehong Prefecture (3)	Kunming-Yuxi (19) Xishuangbanna Dai Autonomous Prefecture-Kunming (17) Kunming-Lincang (9) Baoshan-Kunming (7) Chuxiong Yi Autonomous Prefecture-Kunming (6)
Zhejiang	Hangzhou (10) Ningbo (8) Shaoxing (7) Huzhou (5) Jiaxing (5)	Hangzhou-Shaoxing (99) Hangzhou-Taizhou (88) Hangzhou-Ningbo (81) Hangzhou-Huzhou (51) Hangzhou-Jinhua (51)

## Conclusions and discussions

### Conclusions

This paper analyzes the spatiotemporal evolution of the Chinese urban biomedicine innovation network pattern on four scales, i.e., the national scale, interregional scale, urban agglomeration scale, and provincial scale, using Chinese biomedicine patent cooperation data from the incoPat GPD (2001–2020) and the SNA method to investigate the structure of Chinese urban biomedicine innovation networks. The following conclusions were drawn:

(1) The evolution process of the Chinese national biomedicine innovation network was divided into four phases, namely, the starting phase, growing phase, expanding phase, and mature phase. In all four phases, the network took Beijing and Shanghai as the cores, with its density and complexity continuously improving over time, and its density increased from west to east. Generally, the overall ties in the Chinese national urban biomedicine innovation network have been gradually strengthening, the cooperation scope has been somewhat expanding, and the polarization effect of the network has gradually disappeared. The COVID-19 pandemic has not had an impact on Chinese biomedicine innovation cooperation. In contrast, the numbers of nodes and edges and the centrality and weighted centrality of the network all increased.
(2) On the interregional scale, the interregional urban biomedical innovation networks were superior in both tie closeness and strength to the intraregional urban innovation networks, except for the intraregional urban biomedical innovation network in the eastern region over the 2001–2010 period. Both the centrality of network nodes and the level of intercity biomedicine cooperation in the 2016–2020 period were greatly enhanced compared with those in the 2001–2005 period, with an expansion of the heterogeneous space using regional central cities as cores that formed in the eastern, central and western regions.
(3) The sizes of the urban biomedicine innovation networks among the three major urban agglomerations have evidently increased, and all the cities in the urban agglomerations participated in biomedicine innovation cooperation during the mature phase. The BTH, YRD, and PRD urban agglomerations all developed from single-core urban agglomerations into double-core urban agglomerations. In the interurban-agglomeration biomedicine innovation cooperation, the PRD and the YRD form a double-core urban agglomeration with Guangzhou and Shenzhen as the double cores, while the PRD to BTH and the YRD to BTH networks form single-core urban agglomerations that use Beijing as the core.
(4) On the provincial scale, all intraprovincial biomedicine innovation networks have relatively small sizes, with large variations in average degree, weak connectivity and low internal ties. Each intraprovincial biomedicine innovation network has formed a core-periphery structure with the provincial capital as the center. Excepting Guangdong, Jiangsu and Shandong, the internal ties strengths in all other provinces are lower than 90.

### Improvement and suggestions

(1) It is necessary to fully consider the radiation effects and impetus functions of such central cities as Beijing, Shanghai, Guangzhou, and Shenzhen, and to actively upgrade the function of the edge cities in the entire network through cooperation with the surrounding cities so that they better accept new innovation relationships. A coordinative biomedicine innovation mechanism should be set up to break the communication entanglements and political barriers between cities, promote the integration of intercity biomedicine innovation elements and impair polarization within the network.
(2) Geographical distance plays an important role in the urban biomedicine innovation network. The further the distance between two cities is, the weaker their ties will be. As a result, it is necessary to strengthen the construction of the transportation network in China to lessen the costs of innovation ties between cities.
(3) A government is both an important leader in urban innovation and a formulator of policies, playing a key role in urban biomedicine innovation cooperation. The government should accelerate the implementation of incentive policies regarding biomedicine innovation cooperation and strengthen the protection of property rights within biomedicine. In addition, the government should establish an innovation foundation to increase investment in biomedical innovation.

### Theoretical contribution

(1) This research deals with Chinese urban biomedicine innovation networks on multiple scales, i.e., the national scale, urban agglomeration scale, interregional scale, and provincial scale, by using a three-dimensional method based on the original innovation network research approaches to reveal the pattern characteristics of the innovation networks across different scales. It breaks the limitation of using a single scale on innovation networks and enriches the theoretical system of innovation geography.
(2) Research on urban innovation networks is an important component of research on innovation systems. This research evaluates Chinese urban biomedicine innovation capability on the basis of data related to urban biomedicine patent cooperation, and it contributes to the filed not only by deepening the research on innovation system theory but also by the application of this theory.
(3) This research thoroughly analyzes the positions and functions of various node cities in the innovation networks on the urban agglomeration scale, interregional scale, and provincial scale, etc., and it is conducive not only to enhancing the understanding of the innovative development functions of cities but also to deepening the research on urban geographical theory.

### Shortcomings of research and prospects

(1) This paper studies Chinese urban biomedicine innovation networks solely on the basis of patent cooperation data, which appears to be slightly one-sided for fully describing Chinese biomedicine innovation networks. Next, on the basis of data availability, future research can be conducted with the data related to cooperative papers to comprehensively describe the level of Chinese biomedicine innovation.
(2) Owing to its length, this paper does not analyze the factors influencing Chinese biomedical innovation networks. In future research efforts, the evolution mechanism of the networks can be further explored on the basis of this research.
(3) Considering the research and development of patents requires a certain amount of time, the data set needs to be further expanded in the future. The conclusion made on the basis of an investigation into Chinese biomedicine innovation networks during the 2019–2020 period that found the COVID-19 pandemic has not had a negative impact upon the network needs further testing.

## Data availability statement

The original contributions presented in the study are included in the article/supplementary material, further inquiries can be directed to the corresponding author/s.

## Author contributions

All authors listed have made a substantial, direct, and intellectual contribution to the work and approved it for publication.

## Funding

This work was supported by the Major Program of the National Social Science Foundation of China (Grant Number 20&ZD124).

## Conflict of interest

The authors declare that the research was conducted in the absence of any commercial or financial relationships that could be construed as a potential conflict of interest.

## Publisher's note

All claims expressed in this article are solely those of the authors and do not necessarily represent those of their affiliated organizations, or those of the publisher, the editors and the reviewers. Any product that may be evaluated in this article, or claim that may be made by its manufacturer, is not guaranteed or endorsed by the publisher.

## Footnotes

^1^In calculating patent cooperation, if a patent is applied for jointly by three patent applicants, i.e., A, B, and C, then cooperations between A and B, between A and C, and between B and C will be calculated separately.

^2^The data retrieval date is May 31, 2022.

## References

[1] Salter B Faulkner A. State strategies of governance in biomedical innovation: aligning conceptual approaches for understanding'Rising Powers' in the global context. Global Health. (2011) 7:1–14. 10.1186/1744-8603-7-3 21349182 PMC3049135

[2] Hu F Qiu L Zhou H. Medical device product innovation choices in asia: an empirical analysis based on product space. Front Public Health. (2022) 10:871575. 10.3389/fpubh.2022.871575 35493362 PMC9043244

[3] Feldman MP Francis JL. Fortune favours the prepared region: the case of entrepreneurship and the capitol region biotechnology cluster. Eur Plann Stud. (2003) 11:765–88. 10.1080/0965431032000121337

[4] Chongfeng W Yixuan C. Research on regional independent innovation and open innovation optimization strategy under innovation driving strategy: taking the biomedical industry of China as an example. In: 2018 IEEE International Symposium on Innovation and Entrepreneurship (TEMS-ISIE). Beijing: IEEE (2018). p. 1–10.

[5] Rothwell R. Successful industrial innovation: critical factors for the (1990s). R&D Manag. (1992) 22:221–40. 10.1111/j.1467-9310.1992.tb00812.x

[6] Ter Wal AL. The dynamics of the inventor network in German biotechnology: geographic proximity versus triadic closure. J Econ Geogr. (2014) 14:589–620. 10.1093/jeg/lbs063

[7] Cassi L Plunket A. Research collaboration in co-inventor networks: combining closure, bridging and proximities. Reg Stud. (2015) 49:936–54. 10.1080/00343404.2013.816412

[8] Li Y Phelps NA. Knowledge polycentricity and the evolving Yangtze River Delta megalopolis. Reg Stud. (2017) 51:1035–47. 10.1080/00343404.2016.1240868

[9] Freeman C. Networks of innovators: a synthesis of research issues. Res Policy. (1991) 20:499–514. 10.1016/0048-7333(91)90072-X

[10] Cooke P. The new wave of regional innovation networks: analysis, characteristics and strategy. Small Bus Econ. (1996) 8:159–71. 10.1007/BF00394424

[11] Rycroft RW Kash DE. Complex technology and community: implications for policy and social science. Res Policy. (1994) 23:613–26. 10.1016/0048-7333(94)90012-4

[12] Jones C Hesterly WS Borgatti SP. A general theory of network governance: exchange conditions and social mechanisms. Acad Manag Rev. (1997) 22:911–45. 10.5465/amr.1997.9711022109

[13] Koschatzky K. Innovation networks of industry and business-related services—relations between innovation intensity of firms and regional inter-firm cooperation. Eur Plann Stud. (1999) 7:737–57. 10.1080/09654319908720551

[14] Gardet E Mothe C. SME dependence and coordination in innovation networks. J Small Bus Enterprise Dev. (2012) 19:263–80. 10.1108/14626001211223892

[15] Hu F Xi X Zhang Y. Influencing mechanism of reverse knowledge spillover on investment enterprises' technological progress: an empirical examination of Chinese firms. Technol Forecast Soc Change. (2021) 169:120797. 10.1016/j.techfore.2021.120797

[16] Reagans R McEvily B. Network structure and knowledge transfer: the effects of cohesion and range. Administr Sci Q. (2003) 48:240–67. 10.2307/3556658

[17] Asheim B Coenen L Vang J. Face-to-face, buzz and knowledge bases: socio-spatial implications for learning and innovation policy. Environ Plann C. (2005) 25:655–70. 10.1068/c0648

[18] Ter Wal AL. Cluster emergence and network evolution: a longitudinal analysis of the inventor network in Sophia-Antipolis. Reg Stud. (2013) 47:651–68. 10.1080/00343401003614258

[19] Zhou H Wei S Xi X Zhou H Hu H. Spatiotemporal pattern evolution in global green trade networks: implications for health economics. Discrete Dyn Nat Soc. (2021) 2021:3159747. 10.1155/2021/3159747

[20] Gu W Liu J. Exploring small-world network with an elite-clique: bringing embeddedness theory into the dynamic evolution of a venture capital network. Soc Netw. (2019) 57:70–81. 10.1016/j.socnet.2018.11.002

[21] Henning M McKelvey M. Knowledge, entrepreneurship and regional transformation: contributing to the Schumpeterian and evolutionary perspective on the relationships between them. Small Bus Econ. (2020) 54:495–501. 10.1007/s11187-018-0030-8

[22] Cantner U Graf H Herrmann J Kalthaus M. Inventor networks in renewable energies: the influence of the policy mix in Germany. Res Policy. (2016) 45:1165–84. 10.1016/j.respol.2016.03.005

[23] Engel D Mitze T Patuelli R Reinkowski J. Does cluster policy trigger R&D activity? Evidence from German biotech contests. Eur Plann Stud. (2013) 21:1735–59. 10.1080/09654313.2012.753689

[24] Hu F Qiu L Xia W Liu C-F Xi X Zhao S. Spatiotemporal evolution of online attention to vaccines since 2011: an empirical study in China. Front Public Health. (2022) 10:949482. 10.3389/fpubh.2022.949482 35958849 PMC9360794

[25] Nepelski D De Prato G. The structure and evolution of ICT global innovation network. Industry Innov. (2018) 25:940–65. 10.1080/13662716.2017.1343129

[26] Cassi L Morrison A Ter Wal AL. The evolution of trade and scientific collaboration networks in the global wine sector: a longitudinal study using network analysis. Econ Geogr. (2012) 88:311–34. 10.1111/j.1944-8287.2012.01154.x

[27] Balland P-A De Vaan M Boschma R. The dynamics of interfirm networks along the industry life cycle: the case of the global video game industry, 1987–2007. J Econ Geogr. (2013) 13:741–65. 10.1093/jeg/lbs023

[28] Hu F Xi X Zhang Y Wu R-T. Co-opetition relationships and evolution of the world dairy trade network: implications for policy-maker psychology. Front Psychol. (2021) 11:632465. 10.3389/fpsyg.2020.632465 33603694 PMC7884635

[29] Karna A Täube F Sonderegger P. Evolution of innovation networks across geographical and organizational boundaries: a study of R & D subsidiaries in the B angalore IT cluster. Eur Manag Rev. (2013) 10:211–26. 10.1111/emre.12017

[30] Töpfer S Cantner U Graf H. Structural dynamics of innovation networks in German Leading-Edge Clusters. J Technol Transf. (2019) 44:1816–39. 10.1007/s10961-017-9642-4

[31] Booyens I Hart TG Ramoroka KH. Local innovation networking dynamics: evidence from South Africa. Eur J Dev Res. (2018) 30:749–67. 10.1057/s41287-017-0123-2

[32] Athey G Nathan M Webber C Mahroum S. Innovation and the city. Innovation. (2008) 10:156–69. 10.5172/impp.453.10.2-3.156

[33] Scott J Carrington PJ. The SAGE Handbook of Social Network Analysis. London: SAGE Publications. ISBN: 1847873952 (2011).

